# On the Identification of Elastic Moduli of In-Service Rail by Ultrasonic Guided Waves

**DOI:** 10.3390/s20061769

**Published:** 2020-03-22

**Authors:** Liqiang Zhu, Xiangyu Duan, Zujun Yu

**Affiliations:** 1 School of Mechanical, Electronic and Control Engineering, Beijing Jiaotong University, Beijing 100044, China; lqzhu@bjtu.edu.cn (L.Z.); zjyu@bjtu.edu.cn (Z.Y.); 2 Key Laboratory of Vehicle Advanced Manufacturing, Measuring and Control Technology (Beijing Jiaotong University), Ministry of Education, Beijing 100044, China

**Keywords:** Semi-analytical finite element, Ultrasonic guided waves, Rail, Inverse problem, Material characterization

## Abstract

Non-destructive rail testing and evaluation based on guided waves need accurate information about the mode propagation characteristics, which can be obtained numerically with the exact material properties of the rails. However, for rails in service, it is difficult to accurately obtain their material properties due to temperature fluctuation, material degradation and rail profile changes caused by wear and grinding. In this study, an inverse method is proposed to identify the material elastic constants of in-service rails by minimizing the discrepancy between the phase velocities predicted by a semi-analytical finite element model and those measured using array transducers attached to the rail. By selecting guided wave modes that are sensitive to moduli but not to rail profile changes, the proposed method can make stable estimations for worn rails. Numerical experiments using a three-dimensional finite element model in ABAQUS/Explicit demonstrate that reconstruction accuracies of 0.36% for Young’s modulus and 0.87% for shear modulus can be achieved.

## 1. Introduction

In the process of long-term service, rails will experience deterioration of material properties, fatigue damage, stress concentration, even excessive wear, breakage and other phenomena, which seriously threaten the driving safety. Therefore, the monitoring and detection of rail safety status has always been one of the key points of railway operation and maintenance. Among many testing parameters, the elastic modulus of rail material is one of the parameters that are often examined in rail testing. On the one hand, the change in the elastic modulus itself can directly reflect the decline of rail material and fatigue damage. On the other hand, in many nondestructive testing technologies, it is necessary to know the elastic modulus to establish the mathematical model of rail and obtain the calibration coefficients for testing system. For example, ultrasonic-based monitoring and inspection systems require accurate information regarding wave propagation characteristics, such as wavenumber, phase and group velocity dispersion curves. For rails in service, the actual material and geometric properties can significantly affect the propagation characteristics of wave modes in rails [1]. Hence, this requires evaluating the material elastic properties of the in-service rail for the implementation of guided wave-based monitoring and inspection system under different conditions of rail wear.

Various methods have been proposed to reconstruct the elastic properties of materials. Conventional techniques are destructive in nature, e.g., shear, tensile and compressive tests. As a non-destructive technique, the ultrasonic elastic wave method is more advantageous than conventional techniques. Typically, bulk waves, guided waves or leaky guided waves [2,3,4,5] can be used. In recent years, Lamb wave or guided wave-based techniques have been applied to the inspection of various wave guide structures, such as plates, pipes and rails.

The identification of elastic constants of plates with different materials has been extensively studied. Rogers [5] proposed an inversion method based on a nonlinear least squares method to reconstruct the elastic constants of isotropic plates by measuring the phase velocity and frequency of Rayleigh–Lamb waves. Sale et al. [3] proposed an inverse approach using a simplex search method to estimate the elastic constants of isotropic plates. Group velocity dispersion curves for A0 mode and S0 mode which are extracted using the continuous wavelet transform was employed. Both numerical and experimental validations were presented. In numerical simulations, an estimation errors of shear modulus and Young’s modulus were 1.05% and 0.29%, respectively. The best estimation error of 1.01% was achieved during the experimental validation. Ambrozinski et al. [6] proposed an approach based on guided wave propagation and spatial multiple signal classification to reconstruct the Young’s modulus of an isotropic aluminum plate. Pabisek et al. [4] developed a hybrid computational system for elastic moduli identification for the identification of isotropic plates. An artificial neural network (ANN) was trained in advance and experimental dispersion curves were treated as inputs of the ANN. Yan et al. presented an inversion method of hybrid particle swarm-based-simulated annealing (PS-B-SA) optimization to estimate the elastic properties and thickness of an isotropic thin plate. Webersen et al. [7] also used a simplex search method to determine the elastic constants of orthotropic plate-like materials. Rao [8] and Vishnuvardhan et al. [2] proposed a genetic algorithm (GA)-based approach to reconstruct all nine unknown elastic moduli of orthotropic plates. Eremin et al. [9] proposed an approach to estimate the five effective elastic constants of laminate composite plates using a genetic algorithm. Bochud et al. [10] also used a genetic algorithm to recover the elastic properties of anisotropic plates. An objective function was built from the dispersion equation which allows for accounting for higher-order modes without the need to pair each experimental data point to a specific guided mode. Single transmitter and multiple receivers were employed to obtain the wave velocity of A0 and S0 modes. Rokhlin and Chimenti [11] described a nonlinear inversion scheme to reconstruct the full matrix of elastic constants of orthotropic composite plate using experimental data on reflectivity from a plate immersed in a fluid. Liu et al. [12,13] proposed inversion schemes based on nonlinear least squares and uniform micro-genetic algorithm to determine the material constants of composite laminates and functionally graded material plates.

The identification of the elastic properties of other structures has also been investigated. Karim et al. [14] proposed inversion procedures based on a modified simplex algorithm to invert leaky Lamb wave velocities to estimate the thickness and material elastic constants of an adhesive layer between two aluminum plates and elastic properties of a unidirectional graphite–epoxy composite laminate. Cui et al. [15] proposed a property inversion scheme to reconstruct the elastic moduli of fiber-reinforced composite laminates with a single wave propagation direction and a simulated annealing optimization algorithm with the best accuracy <1% in numerical simulations. Yu et al. [16] proposed an artificial neural network (ANN)-based method to estimate the elastic properties of functionally graded material pipes. The inputs of the ANN model are the group velocities of three fundamental guided circumferential waves at several lower frequencies. More recently, Setshedi et al. [17] proposed an automatic procedure to estimate material and geometric properties of an isotropic homogeneous rail based on the Latin hypercube sampling search strategy. The procedure was developed for an elastic isotropic homogeneous rail and combined elastic moduli, density and frequency as one parameter in the eigenvalue problem. Dispersion curves were computed with different Poisson’s ratio and three geometric parameters. A technique was developed to determine which semi-analytical finite element (SAFE) model best matches the experimental measurements.

So far, most studies on reconstruction of elastic moduli have been aimed at structures with simple geometry like plates and pipes. However, the reconstruction of elastic moduli of structures with complex geometry like in-service rails is not well investigated. Unlike plates or pipes, it is a challenge to select optimal modes and accomplish the excitation and receiving of desired guiding wave modes propagating in rails. What makes the situation more complicated is that, for in-service rail, the cross-section of rail is always changing due to rail wear or rail grinding, which has not been investigated in the literature. In this paper, we proposed an inverse method for the identification of the elastic constants of in-service rail from phase velocities of selected ultrasonic guided wave modes that propagate in specific areas. The forward wave propagation solution here is obtained by the SAFE formulation [18,19,20,21], which is coupled with an improved genetic algorithm (IGA) to match the phase velocities of selected wave modes obtained by the SAFE method to the numerical model which is performed using a commercially available software ABAQUS/Explicit.

It should be noted that the effects of axial load, Young’s modulus and rail wear on the phase velocity of the ultrasonic guided wave in rails had been investigated in our previous studies [18,19,20], where the guided wave modes were selected specifically for estimating the longitudinal axial load of the locked rail experiencing temperature fluctuations, without the prior knowledge of Young’s modulus. In this paper, the focus is on the estimation of elastic moduli, e.g., the Young’s modulus and the shear modulus, in worn rails using guided waves, which has not been studied before. The method proposed in this paper takes into account the sensitivities of different wave modes to the elastic constants and the degree of wear, as well as the single-mode excitation strategy [21,22], in the selection of wave modes. Numerical experiments prove the effectiveness of the method.

The paper is organized as follows. The methodology for the inversion process is presented in Section 2, which consists of the theoretical background of the SAFE formulation and 2-D FFT (two-dimensional fast Fourier transformation) analysis. Modes selection and the excitation method for in-service rail are presented in Section 3. To validate the method, the reconstruction of elastic moduli of 2-meter length worn rails is carried out based on a commercial finite element analysis software ABAQUS/Explicit. The numerical setups and results are described in Section 4. Discussions and conclusions are given in Section 5.

## 2. Methodology for the Inversion Process

### 2.1. SAFE method for Estimating Guided Wave Propagation in Rail

**Figure 1:**
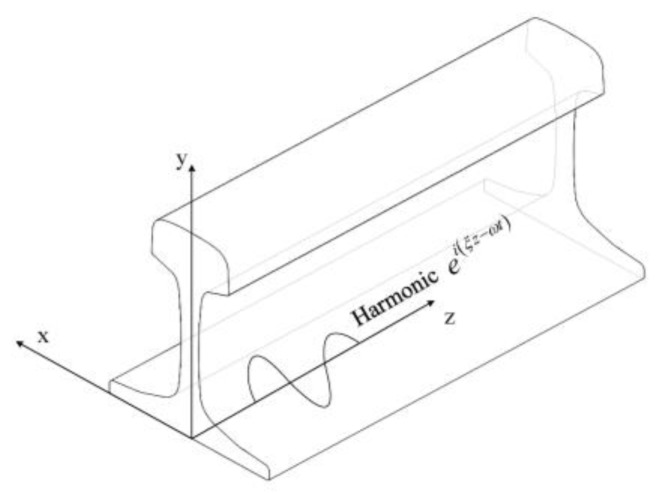
Semi-analytical finite element (SAFE) model of a rail.

The SAFE method is widely used to calculate ultrasonic guided wave propagation solutions for waveguides with a constant cross-section in the wave propagation direction, especially for complex waveguides, e.g., rail [18,22,23,24]. As shown in Figure 1, SAFE treats propagating waves as harmonic waves along the propagating direction, z, and utilizes a two-dimensional mesh of the rail cross-section in plane, (x,y) [20].

According to the virtual work’s principle without considering external forces and traction [25]:

(1) $$\int_{V}\delta u^{T}(\rho \ddot{u})dV+\int_{V}\delta \varepsilon^{T}\sigma dV=0$$

where u, ε and σ are the displacement, strain and stress array, respectively. ρ is the mass density, upper script T indicates a Hermitian matrix, $\ddot{•}$ is the second derivative with respect to time t, and V is the volume.

For an arbitrary point (x,y,z) in the element of a rail, the strain–displacement formulation can be written as:

(2) $$\varepsilon =\left[L_{x}\frac{\partial u}{\partial x}+L_{y}\frac{\partial u}{\partial y}+L_{z}\frac{\partial u}{\partial z}\right]$$

where

(3) $$\begin{array}{l} L_{x}=\left[\begin{array}{ccc} 1 & 0 & 0\\ 0 & 0 & 0\\ 0 & 0 & 0\\ 0 & 0 & 0\\ 0 & 0 & 1\\ 0 & 1 & 0 \end{array}\right],L_{y}=\left[\begin{array}{ccc} 0 & 0 & 0\\ 0 & 1 & 0\\ 0 & 0 & 0\\ 0 & 0 & 1\\ 0 & 0 & 0\\ 1 & 0 & 0 \end{array}\right],L_{z}=\left[\begin{array}{ccc} 0 & 0 & 0\\ 0 & 0 & 0\\ 0 & 0 & 1\\ 0 & 1 & 0\\ 1 & 0 & 0\\ 0 & 0 & 0 \end{array}\right] \end{array}$$

According to the elastic constitutive equation, the stress vector σ can be written as:

(4) σ=Cε

where C is the elastic constant stiffness coefficient matrix. According to Hooke’s law, stiffness matrices for isotropic can be represented by only two independent variables, Young’s modulus E and Passion’s ratio υ [26,27] as

(5) $$C=\frac{E}{\left(1+\upsilon \right)\left(1-2\upsilon \right)}\left[\begin{array}{cccccc} \left(1-\upsilon \right) & \upsilon & \upsilon & & & \\ \upsilon & \left(1-\upsilon \right) & \upsilon & & & \\ \upsilon & \upsilon & \left(1-\upsilon \right) & & & \\ & & & \frac{\left(1-2\upsilon \right)}{2} & & \\ & & & & \frac{\left(1-2\upsilon \right)}{2} & \\ & & & & & \frac{\left(1-2\upsilon \right)}{2} \end{array}\right]$$

It should be noted that the actual rail may not be isotropic, and Matrix C of the the rail under investigation needs to be measured in advance in real applications.

**Figure 2:**
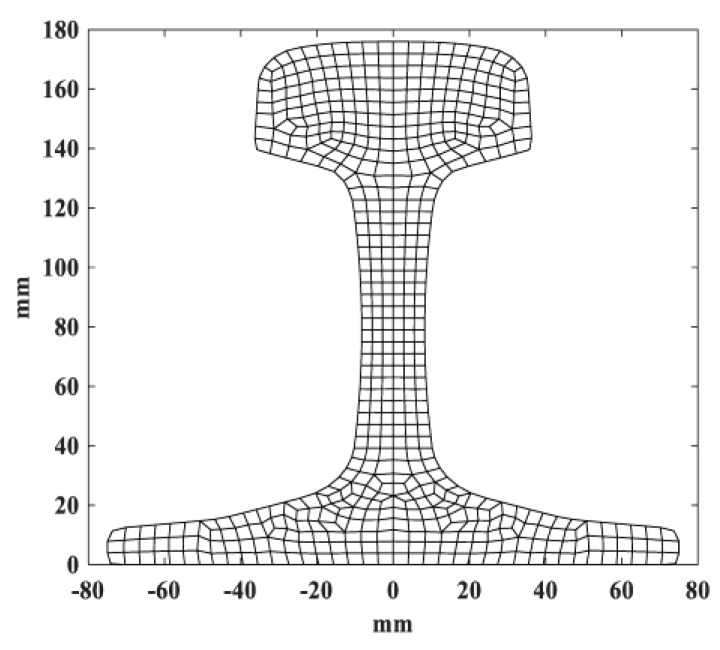
Rail cross-section and sub-divisions.

The rail cross-section can be meshed using triangle or quadratic elements. In this paper, quadratic elements were employed for both SAFE and ABAQUS, as shown in Figure 2. The displacement vector of arbitrary point along the propagating direction is obtained:

(6) $$u=N\left(x,y\right)q^{\left(e\right)}e^{i\left(\xi z-\omega t\right)}$$

where N(x,y) is the shape function matrix depends on the type of mesh element, $q^{\left(e\right)}$ is nodal displacements of arbitrary point, i=sprt(−1) is the imaginary unit, ξ is the wavenumber and ω is the angular frequency.

We substitute Equation (4) and Equation (6) into Equation (1) and apply the standard finite element assembling procedures [20], resulting in:

(7) $$\left(\xi^{2}K_{2}+i\xi K_{1}+K_{0}\right)Q=\omega^{2}MQ$$

where M is the mass matrix,$K_{0}^{},K_{1}^{},K_{2}^{}$ are the stiffness matrix and Q is the global nodal displacement vector.

To eliminate the imaginary unit contained in element stiffness matrix $K_{1}^{}$, a transformation diagonal matrix T is used:

(8) $$T=\left[\begin{array}{ccccccc} i & & & & & & \\ & 1 & & & & & \\ & & 1 & & & & \\ & & & \ddots & & & \\ & & & & i & & \\ & & & & & 1 & \\ & & & & & & 1 \end{array}\right]$$

The final form of eigenvalue equation is obtained:

(9) $$\left(\xi^{2}K_{2}+\xi {\hat{K}}_{1}+K_{0}\right)U=\omega^{2}MU$$

where ${\hat{K}}_{1}=\frac{T^{T}K_{1}T}{-i}$ and U=TQ is a new global nodal displacements vector.

**Figure 3:**
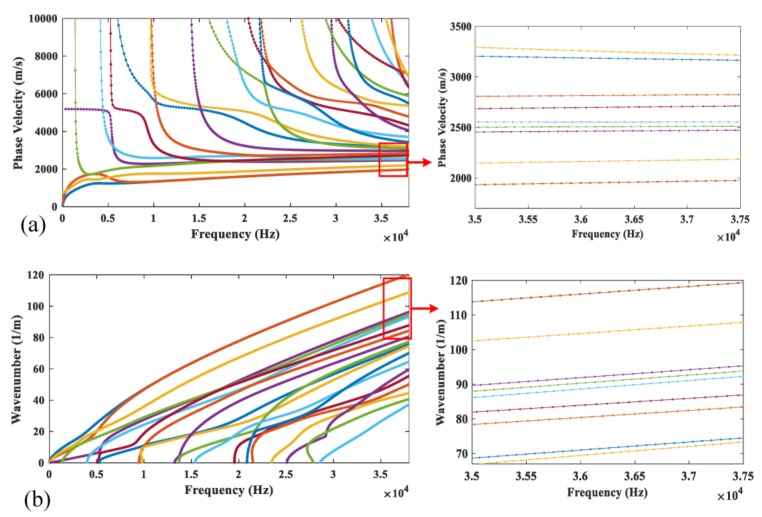
(**a**) Phase velocity and (**b**) wavenumber dispersion curves for CHN60 rail.

The dispersion characteristics of guided waves in rails can be obtained by solving Equation (9). The wave structures of wave modes can also be obtained from the eigenvectors. The phase velocity and wavenumber dispersion curves of CHN60 rail are displayed in Figure 3 with Young’s modulus E=210 GPa, shear modulus G=80.77 GPa, density ρ=700 kg/m^3^ and Poisson’s ratio υ=0.3. The guided wave modes are numbered as mode 1 to mode 10 according to the rule of the phase velocity from small to large (the wavenumber from large to small).

### 2.2. Extraction Method of Phase Velocity

The phase velocities can be obtained by transducer array employing the 2-D FFT method, which was developed by Sachse and Alleyne [28,29]. One needs to acquire an array of time-based waveform with equal space arranged transducers. The 2-D FFT then successively performs temporal and spatial Fourier transformation to transform the time series signal to the frequency-wavenumber domain.

The results of the two-dimensional transformation are an amplitudes matrix corresponding to discrete frequencies and wavenumbers. It should be noted that the spatial resolution should satisfy the sampling criterion and avoid the risks of spatial aliasing. The resolution of the wavenumber depends on the number of transducers and the distance between two transducers. Hanning window function and the zero padded method can be performed to improve the spatial and frequency resolution of the 2-D FFT results. More details of the application of 2-D FFT technique can be found in Ref. [30].

### 2.3. Optimization Approach for Identification of Elastic Constants

The proposed optimization approach estimates the isotropic rail material elastic constants by matching the estimated SAFE-based phase velocities and the measured velocities of selected guided wave mode set. The SAFE-based phase velocities are obtained using the SAFE method, while measured phase velocities are obtained from time series signals by the 2-D FFT.

The genetic algorithm (GA) has been widely used in identifying the elastic moduli [2,9,10]. The common problem occurring in application of GA is premature convergence. The population tends to converge to suboptimal solutions during the process of evolution. To overcome the premature convergence, an improved version of genetic algorithm was used.

Stochastic Universal Sampling (SUS) is used in selection operator. The elitist preservation strategy is also added in selection operator [31,32] to directly copy elitist individual to next generation without performing crossover and mutation operators. The solutions use real-value encoding with arithmetic crossover and uniform mutation. Parent chromosomes are linearly combined during the operation of arithmetic crossover. If the two parent chromosomes are X and Y, then the offspring individuals are $X^{\prime }=rX+\left(1-r\right)Y,Y^{\prime }=rY+\left(1-r\right)X$, where r is a random number between 0 and 1. Uniform mutation means that the individual is replaced by a random individual in the value space. Based on the difference of average and best fitness value of the population (Δ), the adaptive probabilities of crossover $\left(P_{c}\right)$ and mutation $\left(P_{m}\right)$ operators are used, where $k_{1}$ and $k_{2}$ are constants greater than zero [33,34]. The detailed parameters of improved genetic algorithm (IGA) are shown in Table 1.

According to the searching range, a randomly guessed population of elastic constants set is generated first and then all the individuals of population are evaluated using the error function. The error function to be minimized can be represented as follows,

(10) $$ERRf\left(E,G\right)=\sum_{m=1}^{n}\left(\left|\frac{c_{p}^{m,ABAQUS}\left(f\right)-c_{p}^{m,SAFE}\left(f\right)}{c_{p}^{m,SAFE}\left(f\right)}\right|\right)$$

where $c_{p}^{m,SAFE}$ and $c_{p}^{m,ABAQUS}$ are the phase velocities at frequency *f* of *m*-th guided wave mode obtained by SAFE formulation and measured data, respectively.

The evolution process of reconstruction of elastic moduli using IGA can be concluded as follows.

Step 1: Obtain the rail profile J using a two-dimensional laser scanner and phase velocity $c_{p}^{m,SAFE}$ of wave mode set with phase array transducer and 2-D FFT technique, respectively.

Step 2: Generate the initial population according to the parameters used in IGA and searching range for elastic constants.

Step 3: Calculate the phase velocities of selected wave mode set of each individual in the population, using the SAFE model with measured rail profile or standard rail profile.

Step 4: Use error function in Equation (10) to evaluate the fitness of each individual in current generation. If the value of error function is less than the preset tolerance ($toll=10^{-3}$), the iteration will stop and then output the optimal elastic moduli set. Otherwise, proceed to the next step.

Step 5: We compare the fitness value of optimal individual in current population with the optimal individual found in last generation. Directly copy the elite which has the bigger fitness value into the next population.

Step 6: Remaining individuals in the population need to perform crossover and uniform mutation operations. The optimal individual copied from Step 5 and individuals after crossover and mutation form the new population. Then jump to Step 3.

**Figure 4:**
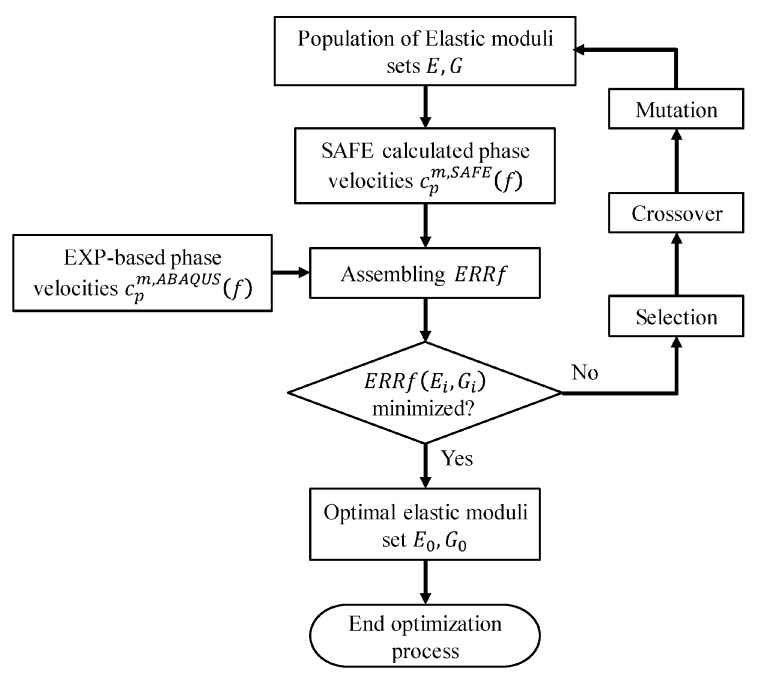
Flowchart of inversion procedures.

The inversion procedures are illustrated in Figure 4.

## 3. Mode Selection and Excitation Method

### 3.1. Mode Selection

Any guided wave modes can be used to reconstruct the elastic moduli of rail, theoretically. However, different modes at 36 kHz have different mode shapes and propagate in different regions of rail cross section with different sensitivities to elastic moduli. In general, for a standard rail profile, one can choose the mode which is most sensitive to elastic moduli. The phase velocities of the first ten wave modes are listed in Table 2 and Table 3, varying with Young’s modulus [35] and shear modulus, respectively. The differences $\Delta c_{p1}$ and $\Delta c_{p2}$ indicate the sensitivities of different modes.

As suggested by the data in Table 2 and Table 3, modes 7, 9 and 10 are more sensitive to elastic moduli and should be selected to reconstruct the elastic moduli of rail, if three modes were to be used. For in-service rail, the profile of in-service rail gradually changes as a result of rail wear or rail grinding, which will change the propagating characteristics of guided wave modes, especially the ones propagating in the rail head. On the other hand, the excitation of wave modes propagating in the rail head is also affected by the changes in the rail profile. Therefore, we need to optimize the criteria in selecting the wave modes used for reconstruction of elastic moduli of in-service rails. For the identification of material elastic constants of in-service rail, there are three aspects we have to take into consideration in selecting an optimal set of wave modes.

First, we should select the wave modes that are sensitive to elastic moduli. An efficient reconstruction will be obtained with these wave modes which have phase velocities that are sensitive to elastic constants. Second, the phase velocities of selected wave modes should be unaffected or less affected by the changes in rail profile in order to design equipment that maintains stable performance for worn rails. Third, the selected wave modes should have relatively simple wave structures which means the excitation and receive are easy to accomplish. It also helps us to get a better accuracy in measuring phase velocities of selected wave modes and improve the accuracy of reconstruction of elastic moduli.

**Figure 5:**
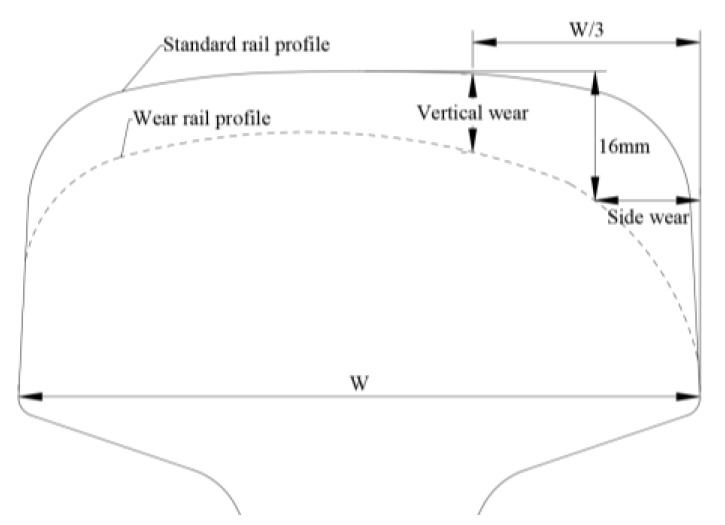
Schematic diagram of rail wear.

Here, we choose three profiles of worn in-service rail to investigate the effect to wave modes caused by rail wear or rail grinding. Side wear and vertical wear are the two main parameters to quantify the rail wear. The rule of measuring the rail wear is shown in Figure 5. The railway regulations defined that vertical wear and side wear are measured at one-third of the width of railhead to the working side and 16mm under the top surface of rail (according to the standard rail profile) [35,36]. The total wear is the sum of half of the side wear and vertical wear.

**Figure 6:**
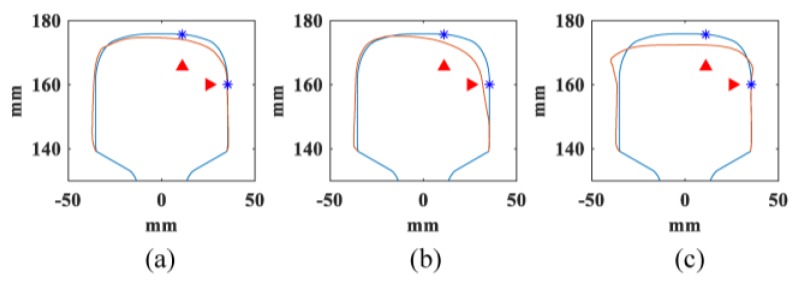
Worn rail profiles of three in-service rails [35].

Three typical rail profiles of worn rails are shown in Figure 6 [35]. Only the profile of the rail head is shown. The standard rail profiles are in blue and worn rail profiles are in red. The asterisk and triangle markers are wear measurement points and the limits of rail wear. The detailed parameters of the three worn profiles are listed in Table 4.

Phase velocities of some guided wave modes of the three worn rails at 36 kHz are obtained using SAFE formulation. The comparison of phase velocities and wavenumber are listed in Table 5 and Table 6. Modes are numbered according to the value of phase velocities. $\Delta cp_{a},\Delta cp_{b},\Delta cp_{c}$ and $\Delta k_{a},\Delta k_{b},\Delta k_{c}$ are the difference (in percent) of the phase velocities and wavenumbers of modes propagating in worn rails to the reference phase velocities and wavenumbers of pristine rails, respectively.

**Figure 7:**
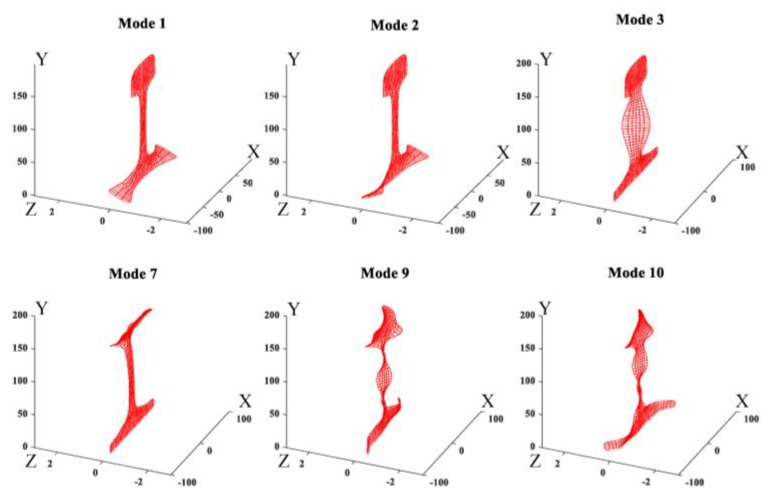
Wave structures of some guided wave modes.

It can be seen from the Table 5 and Table 6 that the phase velocities and wavenumbers of modes 1, 2 and 3 are insensitive to rail wear, while the ones of modes 7, 9 and 10 are slightly changed. The reason behind this phenomenon can be found in the wave structures of these guided modes. By solving Equation (9), we can obtain the wave structures of wave modes. Figure 7 shows the wave structures of wave modes at 36 kHz [35]. As shown in Figure 7, low order wave modes 1, 2, 3 have simple vibration modes that are confined to a specific area of rail web or rail foot and thus insensitive to the rail wear. The vibration mode of high order wave modes 7, 9, 10 are complex and distributed in the whole rail cross-section which is difficult to excite and easily affected by rail wear. From the perspective of equipment design, these low-order modes are also preferred. As their wavenumbers are almost unchanged when the rail profile changes, the arrangement of excitation equipment does not need to change for different rails, especially for the application of phased delay arrays. Therefore, modes 1, 2, and 3 wereused as the optimal set in the inversion procedure. The modes 7, 9 and 10 were chosen as the comparison set. It should be noted that modes 1, 2 and 3 are also less sensitive to a change in elastic moduli when compared to modes 7, 9 and 10.

### 3.2. Excitation Method of Specific Mode

The excitation of desired guided wave modes is the foundation to realize the non-destructive testing of rails. Unlike simple structures like plates and pipes, it is more complicated to excite single specific wave mode in rail. There are many propagating wave modes in rails, especially at high frequencies, as shown in Figure 3 and different cares are needed in exciting and receiving for different wave modes.

**Figure 8:**
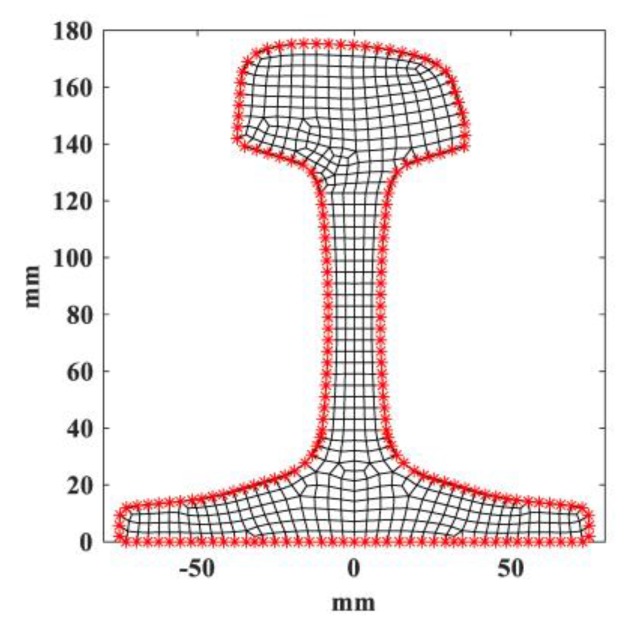
Mesh of worn in-service rail with profile (b) with external nodes marked.

The wave structure contains the displacement information of all nodes of rail cross-section. By analyzing the wave structure information of specific wave modes, we can determine the exact excitation nodes and directions [37]. It is obvious that the guided wave modes can only be excited and received on external nodes of an in-service rail. All the external nodes of a worn in-service rail with profile (b) in the numerical model are marked and shown in Figure 8.

The wave structures of guided wave modes contain displacement information of three directions of different wave modes. We introduce Euclidean distance to represent the difference between wave modes in three dimensional space [38].

The Euclidean distance $Xd_{mn}$ between two wave modes m and n in x direction can be defined as:

(11) $$Xd_{mn}=\sqrt{\sum_{i=1}^{p}{\left(x_{im}-x_{in}\right)}^{2}}$$

where p is the total number of external nodes in the rail profile.

Then, we can get a Euclidean distance matrix to indicate the difference in x direction between wave modes:

(12) $$Xd=\left[\begin{array}{cccc} Xd_{11} & Xd_{12} & \cdots & Xd_{1k}\\ Xd_{21} & Xd_{22} & \cdots & Xd_{2k}\\ \vdots & \vdots & \ddots & \vdots \\ Xd_{k1} & Xd_{k2} & \cdots & Xd_{kk} \end{array}\right]$$

In a similar way, the Euclidean distance matrix in the other two directions can also be calculated with an excitation frequency of 36 kHz. The Euclidean distance indicates the difference between the two modes of vibration in different directions. The greater the Euclidean distance in one direction between the two modes, the more likely it is to get a single mode in this direction.

By comparing the Euclidean distance between the desired wave modes and other wave modes in three directions, the best excitation direction is the one with maximum Euclidean distance.

Then we choose the external nodes that have the maximum positive or negative displacement in the best excitation direction as the excitation nodes, where the positive and negative signs indicate the direction of excitation. For low-order wave modes like mode 1, 2 and 3, which have relatively simple wave structures, we are able to achieve nearly “pure” desired low-order modes by exciting only one or two nodes with the help of phase array excitation. However, for high-order wave modes, more nodes need to be excited to achieve “dominant” desired wave modes. This method is a simplified version of the method proposed by Xu Xining. More details can be found in reference [37].

**Figure 9:**
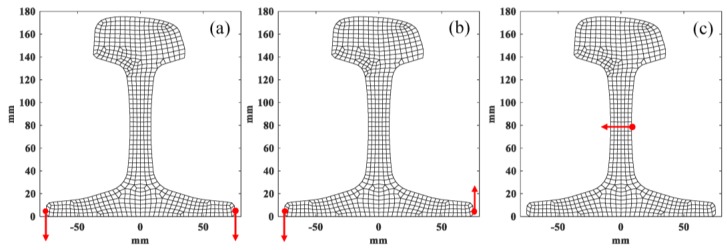
Positions of excitation nodes of (**a**) mode 1 (**b**) mode 2 and (**c**) mode 3.

The best excitation direction and nodes of modes 1, 2 and 3 are obtained using the described excitation method, as shown in Figure 9. Table 7 gives the detail of the excitation of three wave modes. The receiving nodes and direction of each mode are the same as the excitation.

**Figure 10:**
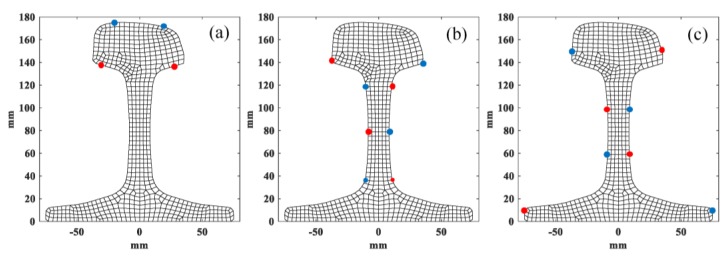
Position of excitation nodes of (**a**) mode 7 (**b**) mode 9 and (**c**) mode 10.

The setup of excitation of mode 7, 9 and 10 was also calculated. It is more complex to excite modes 7, 9 and 10, which need more exciting points due to their complex wave structures. The details of excitation are shown in Table 8 and Figure 10, where red and blue points represent displacements along z positive and negative direction, respectively.

**Figure 11:**
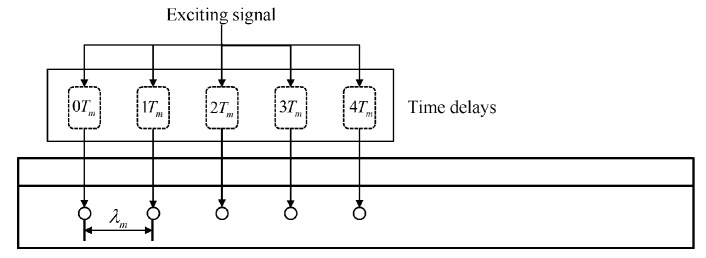
Schematic of phase delay excitation.

In addition, phased delay array excitation along the z direction is implemented based on the comb transducer model proposed by Rose [39], as shown in Figure 11. The phased delay array contains five elements and the spacing between each element is $d=\lambda_{m}$, where $\lambda_{m}$ is the wavelength of the mode m. The time delay of *i*-th element for mode m is

(13) $$Td_{m}=\left(i-1\right)T_{m}$$

where $T_{m}$ is the period of mode m.

## 4. Numerical Validation: Finite Element Analysis

### 4.1. Three-Dimensional Finite Element Model of Continuously Welded Rail

The finite element method has been demonstrated to be efficient in modeling the propagation of elastic guided waves in various structures [3,29,40,41]. Similarly, a commercial finite element package, ABAQUS/Explicit, was used in this study. To ensure the accuracy and stability of numerical results, appropriate element size and time step should be applied. The time step resolution should be satisfied the Courant–Friendrichs–Lewy (CFL) stability criterion [40,41,42]:

(14) $$\Delta t\leq \frac{1}{c_{\max}}\frac{1}{\sqrt{\frac{1}{\Delta x^{2}}+\frac{1}{\Delta y^{2}}+\frac{1}{\Delta z^{2}}}}$$

where $c_{\max}$ is the group velocity of the fastest mode which will propagate in the modelled structure; Δx,Δy,Δz are the dimensions of finite elements.

The size of the finite element should be smaller than one twentieth of the smallest wavelength to be analyzed to get a good spatial resolution:

(15) $$L_{e}=\frac{\lambda_{\min}}{20}$$

where the $\lambda_{\min}$ is the smallest wavelength to be analyzed and $L_{e}$ is the maximum value of the size of the 3-D finite element.

**Figure 12:**
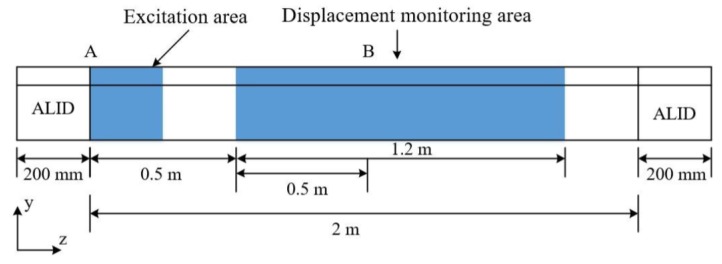
Schematic of the FE setup.

Figure 12 shows the overall FE simulation setup, where a 2-m long worn CHN60 rail was created using C3D8 element and the time step was set as 1ns. The element size in the longitudinal direction z and cross-sectional directions, x and y were 2 mm and less than 4 mm, respectively.

An absorbing layer using increasing damping (ALID) of 200 mm in length was attached to both ends of the rail to absorb waves entering them instead of reflection by the rail end. The ALID consists of a number of layers made of the same material of rail but a gradually increasing damping, and is able to absorb elastic waves propagating into it. The ALID can efficiently suppress the reflected waves and is easily implemented in the commercial finite package [43,44,45].

**Figure 13:**
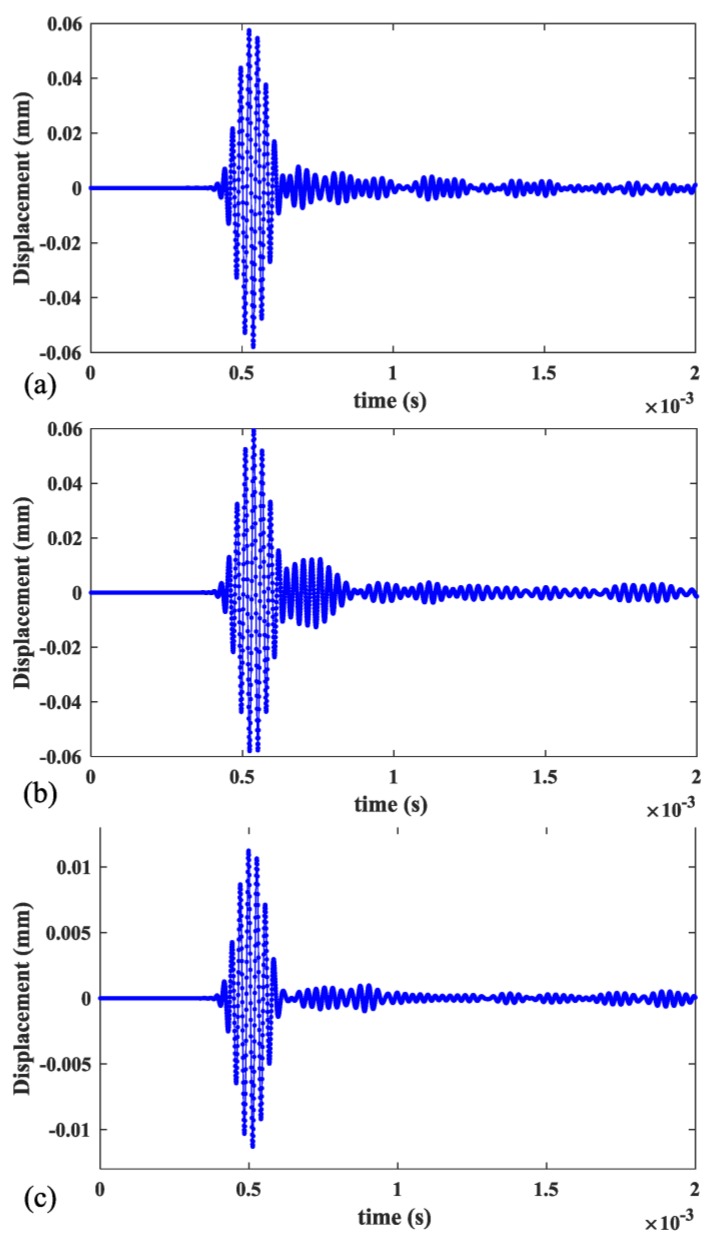
Time history curves of displacement of (a) mode 1, (b) mode 2 and (c) mode 3 at Position B.

**Figure 14:**
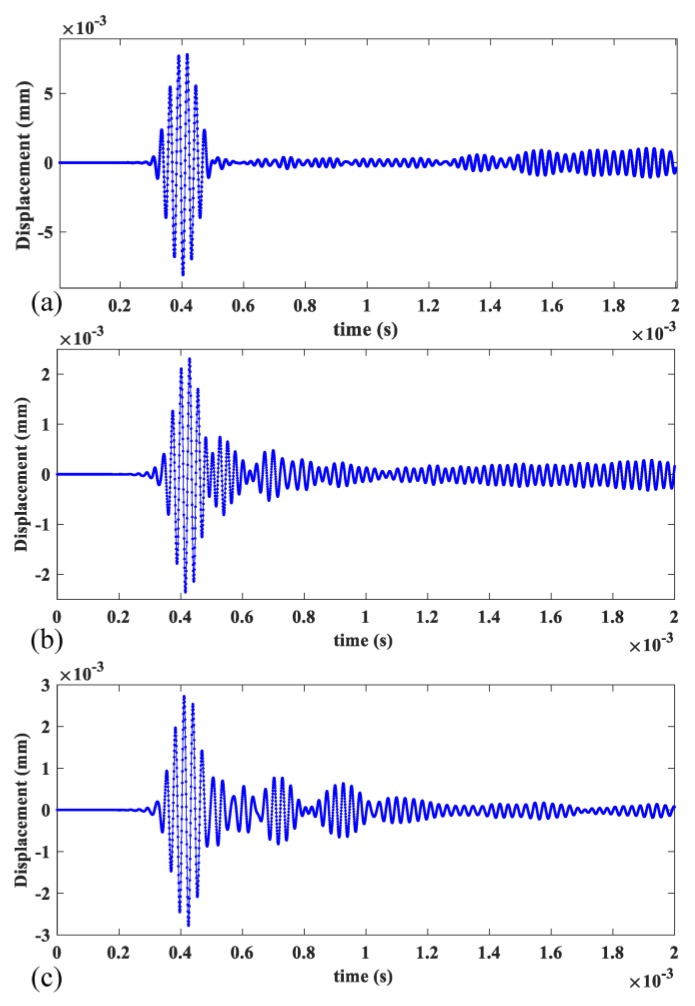
Time history curves of displacement of (**a**) mode 7, (**b**) mode 9 and (**c**) mode 10 at Position B.

The excitation area was located at one end of the rail (Position A in Figure 12) and displacement monitoring area of 1.2 m in length was 0.5 m away from Position A. There were, in total,600 points in the monitoring area. The excitation signal was an 8-cycle Hanning-windowed sinusoidal signal at a center frequency of 36 kHz. The wave mode set containing three wave modes was excited and received with appropriate manners described in Section 3. The displacements occurring at the monitoring area were recorded for measurement of the mode’s phase velocities by applying the 2-D FFT technique. The directions of displacement in Figure 13 are the y direction for mode 1 and mode 2, and the x direction for mode 3 which are decided by the wave structures of these modes. The directions of displacement in Figure 14 are all z direction for mode 7, mode 9 and mode 10 which are also decided by the wave structures of these modes.

To validate the applicability of the proposed method, the worn rail profiles a, b and c, as shown in Figure 6, were employed. The excitation setups can be determined with the method described in Section 3. The following material properties are used: density ρ=7800 kg/m^3^, shear modulus G=81.92 GPa (Poisson’s ratio υ=0.30), Young’s modulus E=213 GPa.

### 4.2. Finite Element Analysis Results

**Figure 15:**
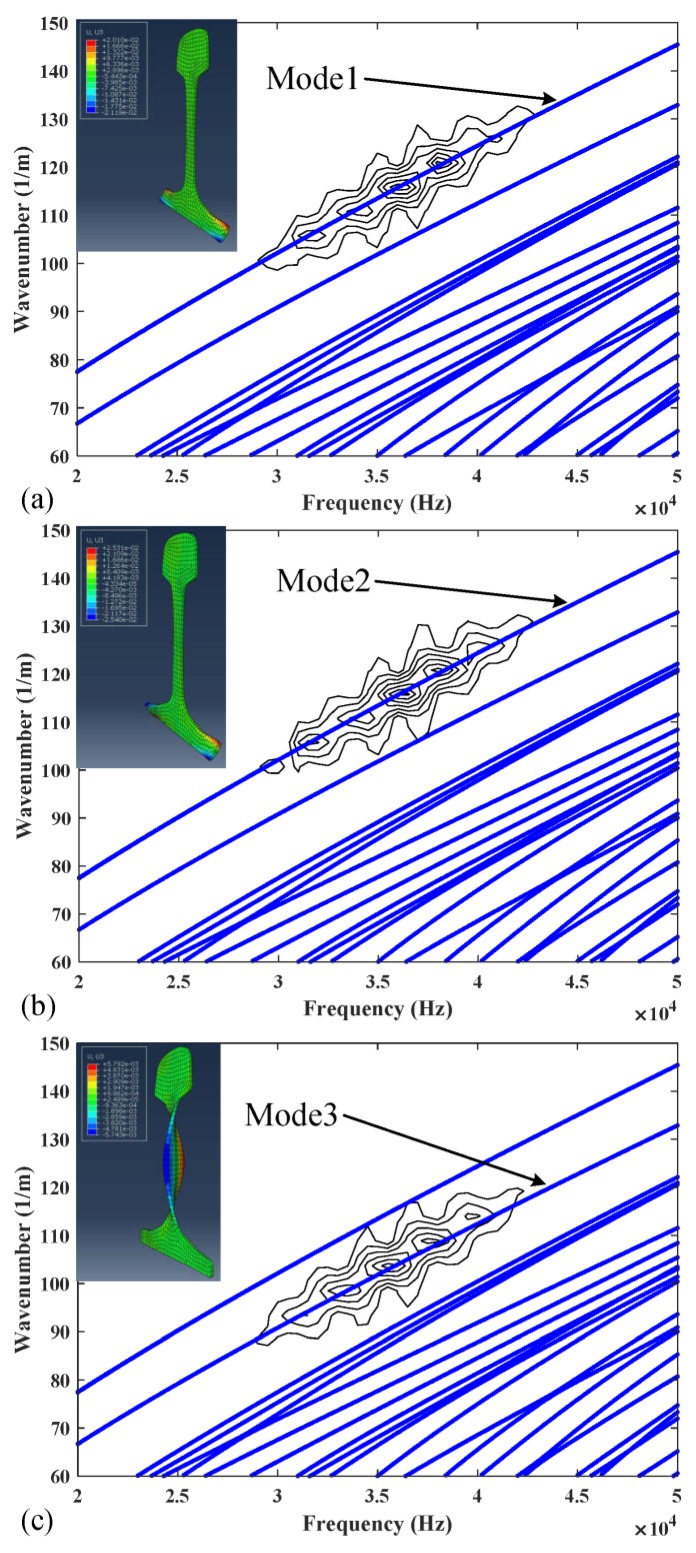
Two-dimensional fast Fourier transformation contours of (**a**) mode 1, (**b**) mode 2 and (**c**) mode 3 with wavenumber dispersion curves superimposed.

**Figure 16:**
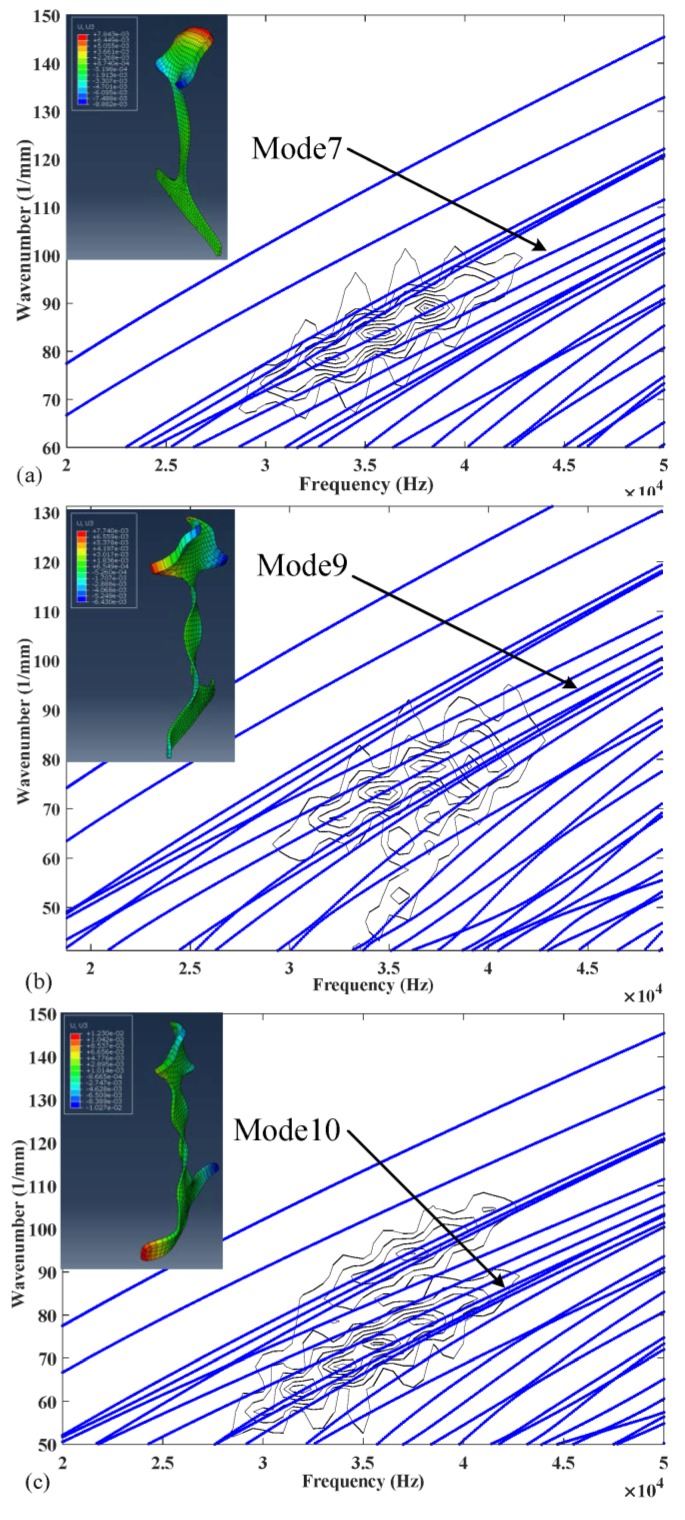
Two-dimensional fast Fourier transformation contours of (**a**) mode 7, (**b**) mode 9 and (**c**) mode 10 with wavenumber dispersion curves superimposed.

When rail profile b is used and modes 1, 2, 3, 7, 9 and 10 are excited individually, the time series of displacements at Position B (1 m away from Position A) are shown in Figure 13a–c and Figure 14a–c respectively. Figure 15 and Figure 16 show the corresponding 2-D FFT contours with wavenumber dispersion curves of rail obtained by SAFE method with same elastic constants superimposed. The lines on the top with a smaller phase velocity represent the low-order guided wave modes. Scalograms of three modes are in accordance with the frequency-wavenumber dispersion curves in Figure 15 and Figure 16, suggesting that the excitation method accomplishes nearly “pure” desired low-order modes and “dominant” desired high-order modes with a ratio of 3.5 in worn in-service rail. The first wave packet in all the time domain waveforms is dominated by the desired wave mode which can be verified from 2-D FFT contours shown in Figure 15 and Figure 16. These modes have similar but not equal group velocity. The differences in group velocities between modes 1, 2, and 3 are less than 60 m/s, and the differences in group velocities between modes 7, 9, and 10 are less than 200 m/s. It is difficult to distinguish these modes in time signals in Figure 15 and Figure 16 because of the propagating distance of these modes in models less than 1 m in length. Moreover, among these modes, mode 1 and mode 2 also have similar phase velocities (the difference in phase velocities is less than 1 m/s). However, without verifying the group velocities of these modes, we can still distinguish them with the wave structures obtained from ABAQUS which are presented in the top left corner of Figure 15 and Figure 16.

Each scalogram provides the amplitude–wavenumber–frequency information of wave modes. The phase velocities can be obtained with the frequency and wavenumber of the scalogram peak with biggest amplitude as follows

(16) $$c_{p}=\frac{2\pi f}{k}$$

where f is the frequency of the wave mode and k is the wavenumber.

### 4.3. Identification of Material Properties Based on the FEM Simulation

The modes phase velocities obtained in Figure 15 and Figure 16 are considered as measured phase velocities. There are two approaches to obtain the SAFE-predicted phase velocities, using standard rail profiles or measured worn rail profiles. It is more convenient to use standard rail profiles, but this usually gets lower precision results than using measured worn rail profiles in practice.

**Figure 17:**
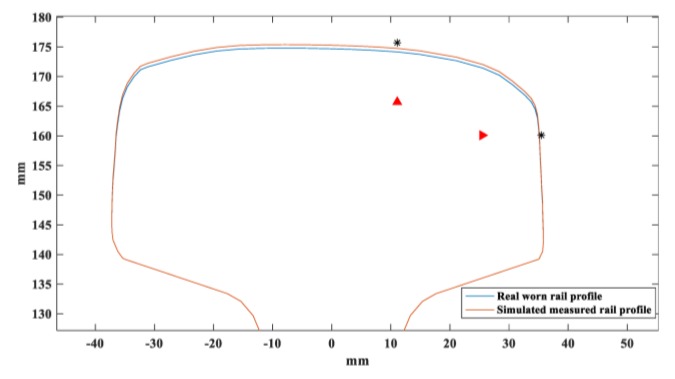
Worn rail profile with simulated measured rail profile.

As for the practical application of in-service rails, the measured rail profile obtained by two-dimensional laser scanning sensors is not exactly the same as the real rail profile. Therefore, a 0.6 mm random deviation from the real rail profile is added to the rail model of SAFE according to the typical accuracy of rail wear gauge, as shown in Figure 17. Furthermore, we also added random white noise to the phase velocities obtained with finite element model to simulate the phase velocity measurement error and the maximum amplitude is 1 m/s.

It should be noted that, except for the rail head, the meshes in the rail web and rail foot of the standard rail profile and worn rail profiles are exactly the same, and the other settings of inversion procedure are also the same.

**Figure 18:**
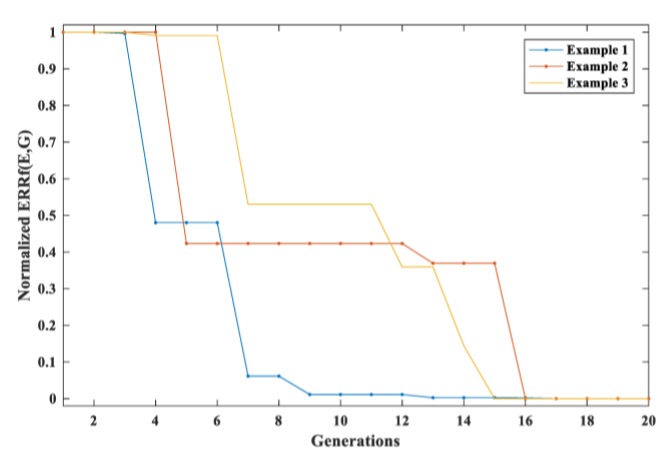
The convergence process of normalized ERRf(E,G) of some examples.

At the beginning of the procedure, a randomly initialized population of elastic moduli set are generated within (206.00~220.00) for Young’s modulus and (71.03~95.65) for shear modulus (0.15~0.45 for Poisson’s ratio). The relative error and standard deviation of the estimated elastic moduli are used to demonstrate the accuracy of the estimation. The IGA method used in this paper is able to coverage within twenty iterations. The convergence process is shown in Figure 18, where different curves are examples from different simulations.

Table 9 and Table 10 show the results of the inversion procedure applied to simulations of three rail profiles with standard and measured rail profile used in SAFE model, respectively. The estimated values are the average of ten runs of GA estimations.

The maximum relative error of reconstruction of Young’s modulus and shear modulus using standard rail profile is <0.69% and <3.14%, respectively. The maximum standard deviation is 0.31 and 0.75 from the mean of elastic moduli, respectively. When the measured rail profile is used, the maximum relative error in shear modulus and Young’s modulus is found to be <0.87% and <0.36%, respectively. The maximum standard deviation is found to be 0.25 and 0.20, respectively.

To see the effectiveness of the optimal mode set consisting of modes 1, 2 and 3, we now consider another mode set to reconstruct the elastic moduli of in-service rail for comparison. Table 11 and Table 12 show the estimation results of the elastic moduli with standard rail profile and measured worn rail profiles using mode 7, 9 and 10, respectively. The estimated values are also averaged over ten estimations.

The maximum relative error of the reconstruction of Young’s modulus and shear modulus using standard rail profile is <1.76% and <9.53%, respectively. The maximum standard deviation is 0.21 and 0.85 from the mean of elastic moduli, respectively. When measured rail profiles are used, the maximum relative error in shear modulus and Young’s modulus is found to be <10.47% and <0.62%, respectively. The maximum standard deviation is found to be 0.81 and 0.21, respectively. The relative errors obtained with standard rail profile are bigger than measured rail profiles.

The optimal mode set we selected obtains a much better performance compared to the mode set consisting of modes 7, 9, 10 in the estimation of elastic moduli using data obtained from numerical simulations. Even if the optimal mode set and standard rail profile in SAFE model are used in the reconstruction process of worn rails, it can make better estimations than the mode set of modes 7, 9 and 10 and measured rail profile in the SAFE model.

## 5. Discussions and Conclusions

An inverse approach to estimate the elastic constants of the material of in-service rails is proposed in this paper. A mode selection method is proposed to achieve a wide applicability for in-service rails which have changing rail profiles due to re-grinding or rail wear. The proposed mode selection approach takes into consideration the influence of the rail profile as well as the excitation and reception of the desired mode which can be realized in practice. The optimal wave mode set, consisting of three low-order wave modes, is selected. Another comparative mode wave set is selected according to the sensitivity to elastic moduli as usual. Comparative numerical studies are carried out to validate the effectiveness and necessity of the mode selection method.

Unlike dispersion curves used in many other studies, the proposed method relies on the measured phase velocities of only three wave modes at one frequency, resulting in more efficient operation in practice. However, on the other hand, the proposed method requires high measurement accuracy of phase velocity. So, the guided wave modes should be carefully selected, and single mode should be excited as purely as possible. There are still some challenges of implementing this strategy in a practical setting. One is the excitation of the pure single mode. The excitation method used in the ABAQUS is based on the displacement imposed on nodes. Due to the size of the real transducer, the results of excitation may be poor. The acquisition of phase velocity of specific modes would be another challenge. The accurate measurement of phase velocity relies on the high resolution of the wavenumber and frequency which need special customized equipments on demand.

The identification of the elastic constants of the materials was achieved through inversion procedures based on an improved genetic algorithm that match the phase velocities of the selected wave mode set obtained from SAFE formulation and numerical simulations. It has to be noted that although the material of in-service rails is treated as isotropic and homogeneous in this paper, the proposed inversion method is suitable for all kinds of materials, as long as the appropriate constitutive relationship of the material can be obtained.

## Figures and Tables

**Table 1:** Parameters used in IGA.

Parameters	Settings
Size of Population	10
Max Generations	20
Selection	Stochastic Universal Sampling and Elitist Preservation
Crossover	Arithmetic crossover
Mutation	Uniform mutation
Probability of Crossover	$P_{c}={\left(1+e^{\left(-k_{1}*\Delta \right)}\right)}^{-1}$
Probability of Mutation	$P_{m}=1-{\left(1+e^{\left(-k_{2}*\Delta \right)}\right)}^{-1}$

**Table 2:** Phase velocities (m/s) variation with different Young’s modulus.

Mode number	E = 212 GPa	E = 216 GPa	$\Delta c_{p1}$
1	1953.916	1969.959	16.043
2	1956.276	1972.344	16.067
3	2205.867	2225.191	19.324
4	2517.632	2544.668	27.036
5	2580.399	2609.510	29.111
6	2651.980	2684.784	32.805
7	2659.204	2686.379	27.175
8	2816.061	2846.475	30.414
9	2970.044	3006.502	36.458
10	3173.780	3222.459	48.679

**Table 3:** Phase velocities (m/s) variation with different shear modulus.

Mode Number	G = 84.0 GPa	G = 81.4 GPa	$\Delta c_{p2}$
1	1936.983	1933.119	3.864
2	1937.294	1933.419	3.875
3	2143.746	2145.276	1.530
4	2463.301	2455.438	7.863
5	2506.153	2501.901	4.252
6	2562.416	2554.812	7.604
7	2711.886	2689.929	21.957
8	2831.896	2811.611	20.285
9	2999.006	2967.924	31.082
10	3152.497	3124.218	28.278

**Table 4:** Details of three worn in-service rails.

Profile Number	Vertical Wear	Side Wear	Total Wear
a	1.50 mm	0.39 mm	1.70 mm
b	2.50 mm	3.66 mm	4.33 mm
c	6.00 mm	5.43 mm	8.72 mm

**Table 5:** Phase velocities (m/s) with different rail profiles.

Mode number	Profile a	$\Delta cp_{a}$ (in percent)	Profile b	$\Delta cp_{b}$ (in percent)	Profile c	$\Delta cp_{c}$ (in percent)
1	1951.8007	0.2115	1951.8007	0.2115	1951.8007	0.2115
2	1952.0716	0.2116	1952.0716	0.2116	1952.0716	0.2116
3	2171.8942	0.1184	2171.8911	0.1183	2171.9765	0.1222
7	2688.0976	0.6833	2689.5073	0.6312	2651.4328	2.0380
9	2957.0585	0.5539	2967.3489	0.2087	2926.0728	1.5960
10	3112.6723	0.2611	3116.1250	0.1504	3108.9001	0.3819

**Table 6:** Wavenumbers (mm) with different rail profiles.

Mode number	Profile a	$\Delta k_{a}$ (in percent)	Profile b	$\Delta k_{b}$ (in percent)	Profile c	$\Delta k_{c}$ (in percent)
1	115.8902	0.2119	115.8902	0.2119	115.8902	0.2119
2	115.8742	0.2121	115.8742	0.2121	115.8742	0.2121
3	104.1463	0.1183	104.1464	0.1181	104.1423	0.1220
7	84.1467	0.6880	84.1026	0.6352	85.3104	2.0804
9	76.4931	0.5570	76.2279	0.2083	77.3032	1.6218
10	72.6690	0.2617	72.5884	0.1506	72.7571	0.3834

**Table 7:** Excitation details of modes 1, 2, and 3.

	Mode 1	Mode 2	Mode 3
Nodes	364, 602	364, 602	674
Direction	y axis negative	y axis positive and negative	x axis negative
Excitation Type	Two-sided symmetric excitation	Two-sided anti-symmetric excitation	Single point excitation

**Table 8:** Excitation details of modes 7, 9, and 10.

	Mode 7	Mode 9	Mode 10
Nodes	198, 233 / 210, 220	231, 342, 674, 251 / 200, 624, 261, 684	603, 266, 679, 202 / 363, 669, 256, 229
Direction	z axis positive and negative	z axis positive and negative	z axis positive and negative
Excitation Type	Four points excitation	Two-sided eight points anti-symmetric excitation	Two-sided eight points anti-symmetric excitation

**Table 9:** Estimated elastic moduli of rail (GPa) and relative errors (%) with standard rail profiles using mode 1, 2, 3.

Rail Profile	a	b	c
E expected value	215.0000	213.0000	214.0000
E estimated value	213.4162	211.7086	214.2204
Relative error	0.6918	0.6063	0.1030
Standard deviation σ	0.0281	0.2852	0.3102
G expected value	79.6296	81.9231	83.5938
G estimated value	77.0549	79.3510	81.8056
Relative error	2.5748	3.1397	2.1391
Standard deviation σ	0.1325	0.1950	0.7457

**Table 10:** Estimated elastic moduli of rail (GPa) and relative errors (%) with measured worn rail profiles using mode 1, 2, 3.

Rail Profile	a	b	c
*E* expected value	215.0000	213.0000	214.0000
*E* estimated value	215.1166	212.2261	213.7712
Relative error	0.0542	0.3633	0.1069
Standard deviation *σ*	0.0790	0.1502	0.2009
*G* expected value	79.6296	81.9231	83.5938
*G* estimated value	79.6635	81.4885	84.3168
Relative error	0.0339	0.5183	0.8649
Standard deviation *σ*	0.0425	0.2447	0.1858

**Table 11:** Rail elastic moduli (GPa) and corresponding errors (%) with standard rail profiles using mode 7, 9, 10.

Rail Profile	a	b	c
E expected value	215.0000	213.0000	214.0000
E estimated value	213.0220	215.5720	217.7630
Relative error	0.9200	1.2075	1.7584
Standard deviation σ	0.0879	0.2060	0.1702
G expected value	79.6296	81.9231	83.5938
G estimated value	72.9987	74.4484	75.6311
Relative error	8.3272	9.1240	9.5254
Standard deviation σ	0.1931	0.8450	0.5368

**Table 12:** Rail elastic moduli (GPa) and corresponding errors (%) with measured worn rail profiles using mode 7, 9, 10.

Rail Profile	a	b	c
E expected value	215.0000	213.0000	214.0000
estimated value	213.6946	211.6890	215.0104
Relative error	0.6072	0.6155	0.4721
Standard deviation σ	0.1163	0.0922	0.2090
G expected value	79.6296	81.9231	83.5938
G estimated value	73.2284	78.0646	74.8453
Relative error	8.0388	4.7099	10.4654
Standard deviation σ	0.8046	0.1264	0.6393
